# Books and Pamphlets Received

**Published:** 1887-10

**Authors:** 


### BOOKS AND PAMPHLETS RECEIVED.

*Elementary Microscopical Technology;* A Manual for Students of Microscopy, in three parts. Part I. The Technical History of a Slide from the Crude Materials to the Mount, by Frank L. James, Ph D., M.D., President St. Louis Society of Microscopists: Member of American Society of Microscopists; Editor St. Louis Medical and Surgical Journal. St. Louis, Mo.; St. Louis Medical and Surgical Journal Co. 1887.

A leading and certainly a very worthy object of this work is to simplify the manipulations and processes demanded in the use of the microscope that the student may be able to understand and avail himself of the facts without a teacher, and without the usual long and arduous labor required in attaining the details of a knowledge of the use of the instrument. The present is a volume of 106 octavo pages and is devoted entirely to elementary technology. It contains only Part I of a series which the author proposes to publish on General Microscopical Technology.

*Transactions of the Medical Association of the State of Missouri,* at its Thirteenth Annual Session, held at Macon City, Missouri, May 10, 1887.

A neat pamphlet of 159 pages, containing the usual reports and discussions.

The following are the officers elect for the ensuing year: President, Frank J. Lutz, St. Louis; Vice-Presidents, T. C. Boulware, Butler; T. B. Jackson, Macon; J. R. Hall, Marshall; W. B. Adams, Montgomery City; J. W. Heddens, St. Joseph; Recording Secretaries, J. C. Mulhall, St. Louis; J. H. Duncan, Kansas City; Corresponding Secretary, W. E. Evans, Boonville; Treasurer, C. A. Thompson, Jefferson City. The time and place of the next meeting is not stated in the book, at least in any place it could be found. It ought always to be put as the last item at the close of the minutes. In most cases it can only be found by rerding the entire proceedings, or as in this case cannot be found at all.

*The Students' Guide to Diseases of the Eye* By Edward Nettleship, F. R.C.S., Ophthalmic Surgeon to St. Thomas' Hospital; Assistant Surgeon to the Royal London Ophthalmic Hospital; Ophthalmic Surgeon to the Hospital for Sick Children, Great Ormond street. Third American from the fourth English edition, with a chapter on examination for color perception, by Wm. Thompson, M.D , Professor of Ophthalmology in the Jefferson Medical College of Philadelphia. Lea Brothers & Co., Philadelphia. 1887.

We have here a work of 475 pages neatly and ably gotten up, in which the author has evidently sought to give us the best and latest upon the department of which he treats. Much new matter has been added from the previous editions of the book. The illustrations are good and a number of new ones have been added. The work will prove useful to the practitioner and is well adapted to students.

---

SANDERS & SONS' EUCALYPTI EXTRACT (*Eucalyptol*).—Apply to Dr. Sander, Dillon, Iowa, for gratis supplied reports on cures effected at the clinics of the Universities of Bonn and Griefswald.

